# Evaluating measures of quality of life in adult scoliosis: a protocol for a systematic review and narrative synthesis

**DOI:** 10.1186/s13643-021-01811-5

**Published:** 2021-09-27

**Authors:** James E. Archer, Charles Baird, Adrian Gardner, Alison B. Rushton, Nicola R. Heneghan

**Affiliations:** 1 The Royal Orthopaedic Hospital NHS Foundation Trust, Bristol Road South, Northfield, Birmingham, UK; 2 Centre of Precision Rehabilitation for Spinal Pain (CPR Spine), School of Sport, Exercise and Rehabilitation Sciences, College of Life and Environmental Sciences, University of Birmingham, Birmingham, UK; 3 School of Physical Therapy, Faculty of Health Sciences, Western University, London, Canada

**Keywords:** Adult scoliosis, Health-related quality of life, Systematic review

## Abstract

**Background:**

Adult scoliosis represents a distinct subgroup of scoliosis patients for whom the diagnosis can have a large impact on their health-related quality of life (HR-QOL). Therefore, HR-QOL patient-reported outcome measures (PROMs) are essential to assess disease progression and the impact of interventions. The objective of this systematic review is to evaluate the measurement properties of HR-QOL PROMs in adult scoliosis patients.

**Methods:**

We will conduct a literature search, from their inception onwards, of multiple electronic databases including AMED, CINAHL, EMBASE, Medline, PsychINFO and PubMed. The searches will be performed in two stages. For both stages of the search, participants will be aged 18 and over with a diagnosis of scoliosis. The primary outcome of interest in the stage one searches will be studies which use PROMs to investigate HR-QOL as defined by the Core Outcome Measures in Effectiveness Trials (COMET) taxonomy, the secondary outcome will be to assess the frequency of use of the various PROMs. In stage two, the primary outcome of interest will be studies which assess the measurement properties of the HR-QOL PROMs identified in stage one. No specific measurement property will be given priority. No planned secondary outcomes have been identified but will be reported if discovered. In stage one, the only restriction on study design will be the exclusion of systematic reviews. In Stage two the only restriction on study design will be the exclusion of full-text articles not available in the English language.

Two reviewers will independently screen all citations and abstract data. Potential conflicts will be resolved through discussion. The study methodological quality (or risk of bias) will be appraised using the Consensus-based Standards for the selection of Health Measurement Instruments (COSMIN) checklist. The overall strength of the body of evidence will then be assessed using the Grading of Recommendations, Assessment, Development and Evaluation (GRADE) approach. A narrative synthesis will be provided with information presented in the main text and tables to summarise and explain the characteristics and findings of the included studies. The narrative synthesis will explore the evidence for currently used PROMs in adult scoliosis patients and any areas that require further study.

**Discussion:**

The review will help clinicians and researchers identify a HR-QOL PROM for use in patients with adult scoliosis. Findings from the review will be published and disseminated through a peer-reviewed journal and conference presentations.

**Systematic review registration:**

This systematic review has been registered with the International Prospective Register of Systematic Reviews (PROSPERO), reference number: CRD42020219437

**Supplementary Information:**

The online version contains supplementary material available at 10.1186/s13643-021-01811-5.

## Background

The International Classification of Functioning, Disability and Health (ICF) aims to provide a framework for describing the complex interaction of both medical and social models of disability and their overall impact on disability [1]. This seeks to conceptualise an individual’s experiences as an interaction between several factors with health conditions forming an important element. Scoliosis is a potentially serious health condition which is characterised by a three-dimensional deformity of the spine [2]. For adult patients, this is defined as a greater than 10-degree curve in the coronal plane using plain film radiographs [3, 4]. Adult scoliosis has a prevalence of between 2.9% and 32% [5–8]. The vast majority of these patients experience back pain, radicular pain, spinal claudication and progression of curvature with cosmesis rarely a cause for presentation for surgical management [4].

Adult scoliosis is considered an umbrella term for two distinct pathological processes, adult idiopathic scoliosis and adult degenerative scoliosis [4]. Adult idiopathic scoliosis has an unknown aetiology, likely a mixture of genetic and mechanical factors [4]. In contrast, adult degenerative scoliosis develops de novo due to progressive degenerative structural changes in the spine normally leading to a loss of lumbar lordosis in the sagittal plane and development of scoliosis [4]. Ultimately both of these conditions lead to a high level of functional disability due to back and leg pain and this can lead to a poor health-related quality of life (HR-QOL). HR-QOL was defined using the World Health Organization definition, ‘An individual’s perception of their position in life in the context of the culture and value systems in which they live and in relation to their goals, expectations, standards and concerns’ [9]. One study, which aimed to assess the impact of chronic conditions on HR-QOL, showed that patients with adult scoliosis had significantly lower scores on the short form-36 assessment than patients with diabetes, chronic lung disease, congestive heart failure or arthritis [10]. This highlights the significant burden this condition can have on HR-QOL when compared to other significant chronic conditions.

The aims of spinal surgery for adult scoliosis patients are to improve HR-QOL by reducing pain from spinal deformity, degenerative change and neural compression [11, 12]. Often younger patients, and those with less significant deformities, are initially managed conservatively [13], however due to the degenerative nature of adult scoliosis, these are rarely effective [14]. The only non-surgical interventions which were found to be effective were the use of non-steroidal anti-inflammatories and activity modification [14]. Health economic evaluations have also been performed to demonstrate the cost-effectivity of surgical interventions for these patients [15].

In the absence of a robustly developed core outcome set, decision making surrounding the measurement of treatment outcomes on HR-QOL can be challenging. What is clear is the importance of well-defined patient-reported outcome measures (PROMs) for patients with scoliosis [16]. The most widely available PROM used to assess HR-QOL in patients with scoliosis, is the Scoliosis Research Society (SRS) questionnaire with its various derivations [17–20]. It has been developed as an effective tool for assessing a wide variety of patients with scoliosis [19], however it has not had its measurement properties tested in discrete sub-populations of patients, such as those with adult scoliosis, to inform practice.

A systematic review which assessed adult scoliosis patients after surgical intervention showed an improvement in HR-QOL at a minimum of 2-year follow-up [21]. However, this study highlighted that there was limited data with which to draw this conclusion and highlighted the need for further work. A systematic review has not been performed on the measurement properties of PROMs in adult scoliosis patients.

Assessment of the measurement properties for PROMs is essential to help reduce bias and ensure accuracy in the results [22]. The consensus-based standards for the selection of health measurement instruments (COSMIN) [22, 23] checklist assess the measurement properties of reliability, validity, interpretability and responsiveness. This methodology allows for an overall assessment of the quality of the PROMs.

The aim of this systematic review is to identify the PROMs used to assess HR-QOL in patients with adult scoliosis and to assess the measurement properties of these PROMs.

## Methods

A systematic review and narrative synthesis will be conducted. This protocol was developed in collaboration with experts in musculoskeletal rehabilitation research and spinal surgery. We used the COnsensus-based Standards for the selection of health Measurement Instruments (COSMIN) methodology and practical tools [22] for selecting the most suitable outcome measurement instruments. The present review protocol is being reported in accordance with the reporting guidance the Preferred Reporting Items for Systematic Reviews and Meta-Analyses Protocols (PRISMA-P) statement [24] (see PRISMA-P checklist in Additional file 1). This review protocol was registered within the International Prospective Register of Systematic Reviews (PROSPERO) (registration number: CRD42020187544). The planned systematic review described in this protocol will be reported following the Preferred Reporting Items for Systematic Reviews and Meta-Analyses (PRISMA) statement [25].

Our protocol reports two separate stages to allow us to fulfil our objectives. In stage one, studies will be identified that use PROMs to investigate HR-QOL in patients with adult scoliosis. From this search, we will obtain a list of PROMs. In stage two we will then assess the measurement properties of the HR-QOL PROMs identified in stage one.

### Stage one: Identifying PROMs of HR-QOL

#### Eligibility criteria

The stage one search aims to identify all PROMs of HR-QOL in adult scoliosis patients and will be selected based on the following criteria: Participants, Outcome and Study design.

#### Participants

Patients aged 18 years and older with a diagnosis of adult scoliosis, as defined by the SRS with a Cobb angle of more than 10 degrees in the coronal plane [26].

#### Outcome

Our primary outcome is to identify any study that includes a PROM of HR-QOL for patients with adult scoliosis. HR-QOL was defined using the World Health Organization definition, ‘An individual’s perception of their position in life in the context of the culture and value systems in which they live and in relation to their goals, expectations, standards and concerns’ [9]. We have defined PROM according to the Core Outcome Measures in Effectiveness Trials (COMET) taxonomy [27]. Our secondary outcome will be to tally the frequency of use of the PROMs.

#### Study design

All study designs (randomised control trials, cohort studies, observational studies and case studies) will be included with the exception of systematic reviews. There will be no limit on language at this stage.

### Stage two: Assessing measurement properties of identified PROMs

#### Eligibility criteria

The stage two search aims to identify all studies assessing the measurement properties of PROMs of HR-QOL in adult scoliosis patients. Studies will be selected based on the following criteria: Participants, Outcome and Study design.

#### Participants

Patients aged 18 years and older with a diagnosis of adult scoliosis, as defined by the SRS [26]. In cases of mixed cohorts, > 50% of the participants should have adult scoliosis. Authors of studies will be contacted in case of missing information about study participants. Systematic reviews will be excluded as they do not contain original patient information.

#### Outcome

The primary outcome will be the measurement properties of PROMs identified in search one. The measurement properties of interest have been selected based on the Delphi study which agreed the measurement properties of interest in HR-QOL PROMs [23] are:

- Reliability
  - Internal consistency
  - Test–retest
  - Inter-rater
  - Intra-rater
  - Measurement error
- Validity
  - Content validity
  - Structural validity
- Interpretability
- Responsiveness of the PROM [23]

No planned secondary outcome measures have been identified; however, if during the review process a point of interest is discovered, this information will be collated and reported.

#### Study design

All studies which evaluate one or more measurement properties of the identified PROMs from stage one will be eligible. This includes any development or validation studies of a PROM. Full-text studies not available in English will be excluded.

### Information sources and search strategy

A comprehensive search strategy will be performed using National Institute for Health and Care Excellence (NICE) Healthcare Databases Advanced Search (HDAS) tool including the following electronic databases: AMED (from inception onwards) CINAHL (from inception onwards), EMBASE (from inception onwards), Medline (from inception onwards), PsychINFO (from inception onwards) and PubMed (from inception onwards)) following consultations with experts and based on scoping searches. Additional specific searches of specialist spinal journals will also be performed. Study authors will be contacted if clarification is required.

An example of the stage one search strategy is included as additional file 1. Multiple uses of the same outcome measure will be tallied.

The stage two search strategy utilises published search strategies [28] and an example is included in additional file 2.

### Data management

Searches will be performed using NICE HDAS tool by two authors (JA and CB). The results will be exported into the Rayyan QCRI [29] tool to allow for removal of duplications. This tool then allows for simultaneous blinded review of the potentially eligible studies by the two reviewers (JA and CB) before unblinding and comparison of decisions. This method will be used for both search one and search two. After the initial screening of the title and abstract, full articles will then be exported to Mendeley (London, UK) and reviewed individually.

### Selection process

A standardised selection process will be performed by two independent authors (JA and CB). In stage one and two, the titles and abstracts will be assessed against the pre-determined selection criteria. Any article where the title and abstract do not provide a clear answer, the full-text article will be retrieved and reviewed. A PRISMA diagram will be constructed [25] to allow transparency over the study flow including reasons for exclusion of studies. Articles will be included if both reviewers agree. In any case of disagreement, this will firstly be discussed between the two authors and any remaining disagreement will then involve the senior author (AG) who will mediate and make a final decision if required.

### Data collection process

Once studies have been identified, two authors (JA and CB) will extract the data from the studies independently and in duplicate. Data will be collected into an ‘overview table’ as suggested by the COSMIN methodology [22]. This table is available as additional file 3. If additional information is required, corresponding authors will be contacted by email.

### Data items

The data will be collected into an overview table, which is available as additional file 3.

The data which we will be collecting relate to the participants, outcome and study design. We will collect data on the participants age, gender and scoliosis subtype to allow for assessment of variation between these characteristics.

Data on the PROMs will be collected to allow comparison of the various PROMs and their characteristics. We will also collect data on the measurement properties of these PROMs from within the studies.

Information on the study design will be collected to allow identification of the important characteristics of the studies which will contribute to our final synthesis of the available evidence.

We will not collect any data on funding sources or additional items. We have not made any pre-planned data assumptions or simplifications.

### Risk of bias in individual studies

Single studies may assess multiple measurement properties and therefore these will be considered separately. For each of the measurement properties, a four point rating of Very good, Adequate, Doubtful or Inadequate will be awarded. The rating will be based on the COSMIN checklist and will be performed independently by two authors (JA and CB) at the level of the study. The two authors will then discuss the outcome and any disagreements will be settled by a senior third author (AG). The agreement between assessors will be reported as a percentage. The overall rating for any given study will be based on its lowest scoring standard [30]. This information will help to inform the synthesis as studies with a doubtful or inadequate rating will impact on the overall assessment of the quality of the evidence.

### Data synthesis

Narrative synthesis aims to explore heterogeneity within primary studies in a descriptive manner rather than statistically. Narrative synthesis can be broken down into four elements [31]:

- Developing a theory of how the intervention works, why and for whom
- Developing a preliminary synthesis of findings of included studies
- Exploring relationships in the data
- Assessing the robustness of the synthesis

Guidance exists to support the PRISMA statement in those circumstances when meta-analysis is not possible [32]. This methodology will be followed for reporting the narrative synthesis.

If studies demonstrate sufficient methodological and clinical similarities, the results will be pooled by measurement property and by PROM. Quantitative pooling will only be performed from data regarding patients with adult scoliosis collected using the same statistical parameters. From scoping searches, the authors anticipate that the data will not be suitable for quantitative pooling as the statistical parameters are not consistent. Therefore, a narrative synthesis of the results will be necessary. There is no plan to assess for meta-bias within this review.

### Confidence in cumulative evidence

The Grading of Recommendations Assessment, Development and Evaluation (GRADE) approach using five factors (Risk of bias, inconsistency, indirectness, imprecision and publication bias) will be used to assess the quality of the evidence and produce a rating of ‘High’, ‘Moderate’, ‘Low’ or ‘Very low’ [33, 34]. As described by the COSMIN methodology, publication bias will not be assessed. In the case of inconsistency, and where results cannot be pooled or summarised, the conclusion will be based upon the consistent results which make up the majority. However, the quality of evidence will be downgraded for inconsistency.

## Discussion

The ICF framework recognises the important role that both medical and social models have on an individual patient’s disability. Part of the impact of adult scoliosis on disability can be assessed using HR-QOL PROMs and help to define the long-term impact of this condition. An array of PROMs are utilised for assessing HR-QOL in patients with scoliosis; however, these are not specific to the discrete population subgroups. The impact on HR-QOL is clearly different between adolescent and adult populations [35], with adult scoliosis having a significant impact on patients HR-QOL [10]. While evidence exists that surgery may improve HR-QOL in adult scoliosis patients [21], the measurement properties of the PROMs that are utilised has not been assessed.

It is therefore important that in this subpopulation, PROMs with sufficient measurement properties are available to assess the HR-QOL and evaluate the longitudinal impacts of the condition on patients, the progression of the disease, the impact of management, including surgical correction and to help guide further research advancements.

Clinicians recognise the important role of surgical management in these patients; however, to fully understand the impact of treatments on HR-QOL in patients, PROM with established measurement properties that measure and record the outcomes of management are essential. This systematic review aims to retrieve the PROM that are currently utilised in this patient population and will then evaluate and synthesise the quality of these PROM with respect to their measurement properties. Findings from this review will benefit clinicians in deciding how best to assess the impact of adult scoliosis on patients HR-QOL and guide further research and validation of HR-QOL PROM within this specific subgroup of patients.

### Limitations

The limitations of this review at a study level will be that contacting authors to access full data may be challenging and potentially impact upon the results.

At a review level, the main limitation is likely to be the small volume of eligible studies which exist.

### Protocol amendments

Any amendment that is made to the protocol will be clearly detailed in the publication of the results of the systematic review.

### Patient and public involvement

The study was conceived from our working with patients, including feedback from our PPI group at the Centre of Precision Rehabilitation for Spinal Pain. The specific research question was generated by an expert panel consisting of musculoskeletal and spinal surgical experts with vast experience working with adult scoliosis patients. No patient and public involvement has been included in data collection as no new data collection is required. Patient representatives may be involved as necessary to support interpretation of findings and co-write the plain English summary.

### Ethics and dissemination statement

This systematic review does not require ethical approval. All results of this review will be published in peer-reviewed journals and presented at our PPI group, national and international conferences.

## Supplementary Information

**Additional file 1.** Appendix 1 – Search strategy one.

**Additional file 2.** Appendix 2 – Search strategy two.

**Additional file 3.** Appendix 3 – Data collection table.

## Abbreviations

HR-QOL: Health-related quality of life
PROM: Patient-reported outcome measure
PRISMA: Preferred Reporting Items for Systematic Reviews and Meta-Analysis
COSMIN: Consensus-based Standards for the Selection of health Measurement Instruments
GRADE: Grading of Recommendations, Assessment, Development and Evaluation
ICF: International Classification of Functioning, disability and health
SRS: Scoliosis Research Society
PROSPERO: International Prospective Register Of Systematic Reviews
COMET: Core Outcome Measures in Effectiveness Trials
NICE: National Institute for Health and Care Excellence
HDAS: Healthcare Databases Advanced Search
