# Supplementary material for: Evaluating measures of quality of life in adult scoliosis: a protocol for a systematic review and narrative synthesis
Source: Syst Rev. 2021 Sep 27;10:259. doi: 10.1186/s13643-021-01811-5 (PMC8474779; doi:10.1186/s13643-021-01811-5)
Supplement: Supplementary file 1 — Additional file 1. Appendix 1 – Search strategy one. [file 13643_2021_1811_MOESM1_ESM.docx]

**Appendix 1 – Search strategy one**

adult scoliosis OR degenerative scoliosis OR adult degenerative scoliosis OR adult idiopathic scoliosis

AND

Quality of Life

OR quality of life

OR life qualit*

OR living qualit*

OR quality of living

OR Activities of Daily Living

OR activities of daily living

OR activity of daily living

OR activities of daily life

OR activity of daily life

OR daily living activit*

OR daily life activit*

OR adl

OR chronic limitation of activity

OR self care*

OR Health Status

OR health status

OR level of health

OR health level*

OR qol

OR hrql

OR hrqol

OR activity of daily living

OR activities of daily life

OR activity of daily life

OR daily life activit*

OR iadl

OR living qualit*

OR quality of living

OR Activities of Daily Living

OR adl

OR activities of daily living*

OR daily living activit*

OR limitation of activit*

OR independent living*

OR iadl*

OR everyday function*

OR functional abilit*

OR daily function*

OR physical function

OR physical function*
