# Supplementary material for: Evaluating measures of quality of life in adult scoliosis: a protocol for a systematic review and narrative synthesis
Source: Syst Rev. 2021 Sep 27;10:259. doi: 10.1186/s13643-021-01811-5 (PMC8474779; doi:10.1186/s13643-021-01811-5)
Supplement: Supplementary file 2 — Additional file 2. Appendix 2 – Search strategy two. [file 13643_2021_1811_MOESM2_ESM.docx]

**Appendix 2 – Search strategy two**

PROM (identified from search one)

AND

adult scoliosis

OR degenerative scoliosis

OR adult degenerative scoliosis

OR adult idiopathic scoliosis

AND

validity

OR validation studies

OR reliability

OR reproducibility of results

OR interpretability

OR internal consistency

OR clinical sensitivity

OR psychometrics

OR responsiveness

OR evaluation studies

OR measurement error

OR measurement properties
