# Supplementary material for: Evaluating measures of quality of life in adult scoliosis: a protocol for a systematic review and narrative synthesis
Source: Syst Rev. 2021 Sep 27;10:259. doi: 10.1186/s13643-021-01811-5 (PMC8474779; doi:10.1186/s13643-021-01811-5)
Supplement: Supplementary file 3 — Additional file 3. Appendix 3 – Data collection table. [file 13643_2021_1811_MOESM3_ESM.docx]

**Appendix 3 – Data collection table**

| PROM | Year of  development | Construct | Target population | Mode of administration | Recall period | Subscales (number of items) | Response options | Range of scores | Original language | Available translations | No. of  evaluation studies |
| --- | --- | --- | --- | --- | --- | --- | --- | --- | --- | --- | --- |
|  |  |  |  |  |  |  |  |  |  |  |  |
|  |  |  |  |  |  |  |  |  |  |  |  |
